# Production of rhamnolipid biosurfactants in solid-state fermentation: process optimization and characterization studies

**DOI:** 10.1186/s12896-022-00772-4

**Published:** 2023-01-24

**Authors:** Shima Dabaghi, Seyed Ahmad Ataei, Ali Taheri

**Affiliations:** 1 Department of Chemical Engineerig, Faculty of Engineering, Shahid Bahonar University of Kerman, Kerman, Iran; 2 Fisheries Department, Faculty of Marine Sciences, Chabahar Maritime University, Chabahar, Iran

**Keywords:** Biosurfactant, Optimization, Response surface methodology, Solid-state fermentation, Agro-industrial residues

## Abstract

**Background:**

Rhamnolipids are a group of the extracellular microbial surface-active molecules produced by certain *Pseudomonas* species with various environmental and industrial applications. The goal of the present research was to identify and optimize key process parameters for Pseudomonas aeruginosa PTCC 1074s synthesis of rhamnolipids utilizing soybean meal in solid state fermentation. A fractional factorial design was used to screen the key nutritional and environmental parameters to achieve the high rhamnolipid production. Response surface methodology was used to optimize the levels of four significant factors.

**Results:**

The characterization of biosurfactant by TLC, FT-IR and H-NMR showed the rhamnolipids presence. In the optimum conditions (temperature 34.5 °C, humidity 80%, inoculum size 1.4 mL, and glycerol 5%), the experimental value of rhamnolipid production was 19.68 g/kg dry substrate. The obtained rhamnolipid biosurfactant decreased water's surface tension from 71.8 ± 0.4 to 32.2 ± 0.2 mN/m with a critical micelle concentration of nearly 70 mg/L. Additionally, analysis of the emulsification activity revealed that the generated biosurfactant was stable throughout a broad pH, temperature, and NaCl concentration range.

**Conclusions:**

The current study confirmed the considerable potential of agro-industrial residues in the production of rhamnolipid and enhanced the production yield by screening and optimizing the significant process parameters.

## Background

Biosurfactants are a group of secondary metabolites which can reduce the interfacial tension of different phases with various degrees of polarities and hydrogen bonds in terms of their amphiphilic nature, as well as hydrophobic and hydrophilic moieties [1, 2]. Various microorganisms, including bacteria, fungi, and yeasts, have the ability to produce these molecules during their stationary growth [3, 4]. The biosurfactants are divided into many classes based on their chemical makeup and microbiological source, including glycolipids, phospholipids, lipopeptides, natural lipids, polymeric surfactants, and particulate surfactants [5–9]. The glycolipid biosurfactants "rhamnolipids" produced by certain *Pseudomonas* species stand apart in terms of their good emulsification potentials, remarkable physicochemical properties, low toxicity, and antimicrobial activities [10]. The aforementioned advantages made them interesting compounds for the application in a broad range of different areas, such as Bioremediation, enhanced oil recovery (EOR), pharmaceuticals, cosmetics, and the food industry as a new class of renewable resources-based surfactants. [11, 12]. Next to the sophorolipids, which can be found in some cleansing agents, rhamnolipids are most likely the next generation of biosurfactants that will reach the market [13].

However, it is necessary to overcome several limitations, including low efficiency of production, high substrate cost, and considerable costs of separation and purification processes to produce biosurfactants on a large scale [5]. Using the agro-industrial residues and wastes known as biomass and enhancement of production efficiency by screening and optimizing of effective parameters can be efficient [14, 15]. Agro-industrial residues are given economic value by being used to make value-added goods like biosurfactants, which solves the issues with the environment and trash disposal [16, 17]. Many researchers used different types of biomasses for the rhamnolipids production using submerged fermentation (SMF) [18–20]. Nevertheless, a large amount of foam is produced in this method, increasing contamination risk and reducing process productivity [21, 22]. The production of biological products using solid state fermentation (SSF), which involves the growth of microorganisms on a solid substrate in the absence of free water, eliminates the problem of foam formation [10]. Thus, value-added products can be produced with lower costs, higher water and energy storage, and a decrease in the waste and wastewater produced [23, 24]. There are few reports on the production of rhamnolipids by solid-state fermentation. Table 1 shows previous reports of rhamnolipids production in SSF using agro-industrial residues.

**Table 1:** Previous reports of rhamnolipids production in SSF using agro-industrial residues

Substrate	Microorganism	Incubation time	Rhamnolipids production (g/Kg dry substrate)	References
Rice straw and sunflower seed meal	Pseudomonas aeruginosa UFPEDA 614	288 h	25.5	[14]
Corn bran	Pseudomonas aeruginosa UFPEDA 614	288 h	14.5	[14]
Sugarcane bagasse	Pseudomonas aeruginosa UFPEDA 614	288 h	20	[25]
Sugarcane bagasse and sunflower seed meal	Pseudomonas aeruginosa 15GR	10 days	63.3	[26]
Mahua oil cake	Serratia rubidaea SNAU02	7 days	–	[27]

Screening and optimization of significant process parameters are the effective approaches for the enhancement of bioprocess yield. One of the most practical techniques to screen the main factors from a large number of variables considering interaction effects among them is fractional factorial design (FFD) which involves running the partial number of a full factorial design [28]. Response surface method (RSM), a multivariate statistical technique, has long been used in chemical engineering and agro-biotechnology to enhance the outcomes of initial screening as important factors [29]. RSM combines mathematical and statistical techniques to evaluate significant factors' effect which allows the optimization to be effectively conducted [30]. The rhamnolipid production from soybean meal as the renewable carbon source by *Pseudomonas aeruginosa* (PTCC 1074) under solid state fermentation was studied for the first time. A two-step experimental design procedure using FFD and RSM was used to screen and optimize different nutritional and environmental parameters to improve the efficiency.

## Results and discussion

### The production kinetics of rhamnolipids

**Fig. 1:**
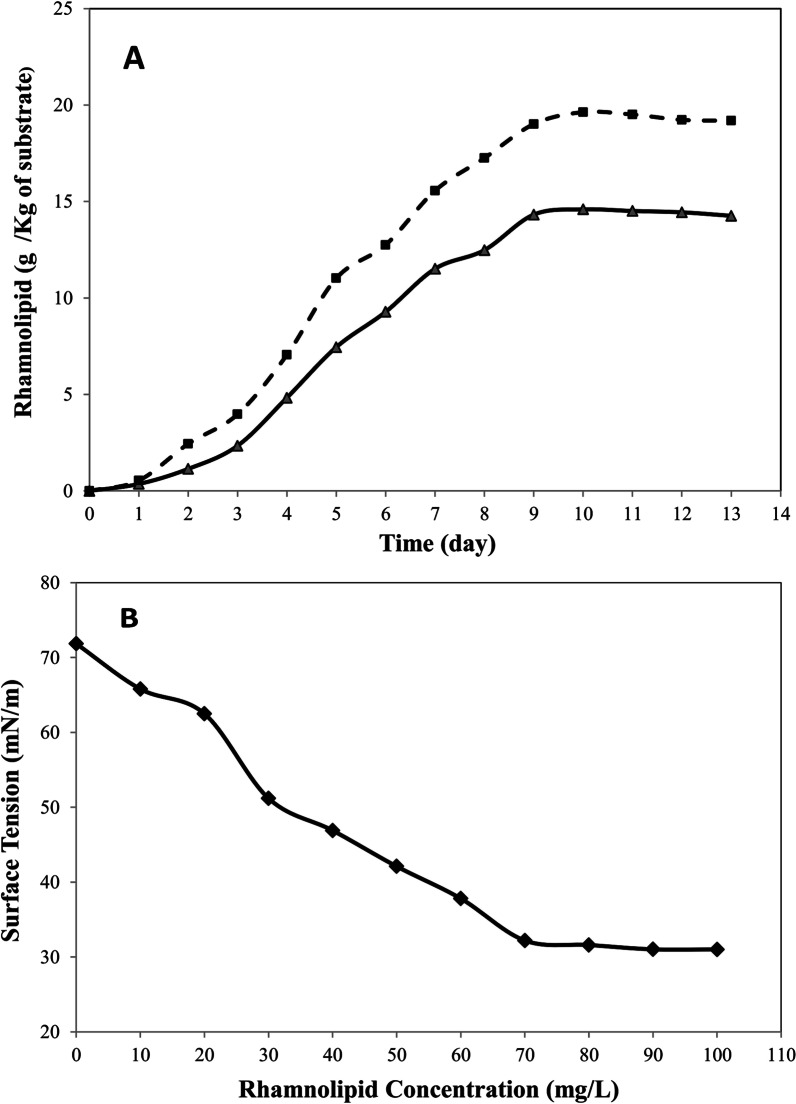
**A** Kinetics of rhamnolipids production: non-optimal conditions (solid line—▲— and optimal conditions (dash line–––), **B** Surface tension values (mN/m) versus rhamnolipid concentration (mg/mL)

In the first step, the production kinetics of rhamnolipids from Pseudomonas aeruginosa PTCC 1074 on soybean meal with an initial humidity of 70% inoculated by 1 mL of inoculum at the temperature of 30 °C was investigated. As can be observed in Fig. 1A, rhamnolipid production linearly increased with the increase in process time to 10 days and reached 14.63 (g/kg substrate), after which it remained constant. Therefore, the duration of 10 days was considered for the completion of fermentation process.

### Screening of significant variables by FFD

The fractional factorial design enables the identification of variables which have a significant role in rhamnolipid production. The FFD experimental findings (Table 2) showed a broad range in biosurfactant generation. Since these variations made interpretation of the findings difficult, screening the key process parameters to achieve higher production was important. A first-order model represented rhamnolipid production as the response variable (y) in terms of coded factors as a function of eight independent variables which were pH (A), concentration of glycerol (B), amount of MgSO_4_.7H_2_O (C), humidity (D), temperature (E), size of substrate (F), inoculum size (G) and amount of NaNO_3_ (H).

1 $$y^{{}} = 13.07 + 0.38A + 0.91B - 0.02C + 1.75D + 1.84E + 0.032F + 1.67G - 0.070H$$

**Table 2:** FFD design matrix for variables with coded values along with the experimental and predicted responses

A: pH	B: Glycerol (%)	C: MgSO_4_ (gr)	D: Humidity (%)	E: Temperature (°C)	F: Particle size (nm)	G: Inoculum size (mL)	H: NaNO_3_ (gr)	Rhamnolipid (g/kg of substrate)	
								**Experimental**	**Predicted**
9.00 (1)	6.00 (1)	0.40 (1)	80.00 (1)	35.00 (1)	1.50 (1)	1.50 (1)	8.00 (1)	18.04	18.94
5.00 ( − 1)	2.00 ( − 1)	0.40 (1)	80.00 (1)	35.00 (1)	0.50 ( − 1)	0.50 ( − 1)	8.00 (1)	14.36	14.37
5.00 ( − 1)	2.00 ( − 1)	0.40 (1)	50.00 ( − 1)	35.00 (1)	1.50 (1)	1.50 (1)	4.00 ( − 1)	14.37	14.21
9.00 (1)	2.00 ( − 1)	0.40 (1)	50.00 ( − 1)	25.00 ( − 1)	1.50 (1)	0.50 ( − 1)	8.00 (1)	7.87	7.20
5.00 ( − 1)	6.00 (1)	0.40 (1)	50.00 (1)	25.00 ( − 1)	1.50 (1)	0.50 ( − 1)	4.00 ( − 1)	11.93	11.93
9.00 (1)	6.00 (1)	0.20 ( − 1)	80.00 (1)	25.00 ( − 1)	0.50 ( − 1)	0.50 ( − 1)	8.00 (1)	12.67	11.92
5.00 ( − 1)	6.00 (1)	0.40 (1)	50.00 ( − 1)	25.00 ( − 1)	0.50 ( − 1)	1.50 (1)	8.00 (1)	12.31	11.77
9.00 (1)	2.00 ( − 1)	0.20 ( − 1)	50.00 ( − 1)	35.00 (1)	0.50 ( − 1)	1.50 (1)	8.00 (1)	14.81	14.21
5.00 ( − 1)	2.00 ( − 1)	0.20 ( − 1)	80.00 (1)	25.00 ( − 1)	1.50 (1)	1.50 (1)	8.00 (1)	13.3	14.04
5.00 ( − 1)	2.00 ( − 1)	0.20 ( − 1)	50.00 ( − 1)	25.00 ( − 1)	0.50 ( − 1)	0.50 ( − 1)	4.00 ( − 1)	5.66	7.20
5.00 ( − 1)	6.00 (1)	0.20 ( − 1)	50.00 ( − 1)	35.00 (1)	1.50 (1)	0.50 ( − 1)	8.00 (1)	10.67	12.10
9.00 (1)	6.00 (1)	0.40 (1)	50.00 ( − 1)	35.00 (1)	0.50 ( − 1)	0.50 ( − 1)	4.00 ( − 1)	12.15	12.10
5.00 ( − 1)	6.00 (1)	0.20 ( − 1)	80.00 (1)	35.00 (1)	0.50 ( − 1)	1.50 (1)	4.00 ( − 1)	18.97	18.94
9.00 (1)	2.00 ( − 1)	0.20 ( − 1)	80.00 (1)	35.00 (1)	1.50 (1)	0.50 ( − 1)	4.00 ( − 1)	15.91	14.37
9.00 (1)	2.00 ( − 1)	0.40 (1)	80.00 (1)	25.00 ( − 1)	0.50 ( − 1)	1.50 (1)	4.00 ( − 1)	13.4	14.04
9.00 (1)	6.00 (1)	0.20 ( − 1)	50.00 ( − 1)	25.00 ( − 1)	1.50 (1)	1.50 (1)	4.00 ( − 1)	12.76	11.77

The adequacy of regression models obtained to fit the experimental data was tested by analyzing variance (ANOVA), and the coefficient of determination (R^2^). ANOVA showed that the obtained model from FFD with F-value of 37.67and P-value < 0.0001 is significant. The value of 0.9320 for R^2^ ensured a good agreement of the first- order model and the experimental data. The humidity, temperature, and inoculum size with P-values < 0.0001 and glycerol concentration with P-value of 0.0333 strongly affected rhamnolipid production. Meanwhile, all other variables with P-values > 0.05 had no significant effect on response. By eliminating the parameters which have no significant effect, the model took the form bellow:

2 $$y^{{}} = 13.07 + 0.91B + 1.75D + 1.84E + 1.67G$$

### The method of steepest ascent for determining optimum region

The coefficient of significant variables (B, D, E, and G) was positive because of the first-order model shown in Eq. (6); this indicates that increasing glycerol concentration, humidity, temperature, and inoculum size had a positive impact on the formation of rhamnolipids. The steepest ascent experimental design and corresponding responses were presented in Table 3. The mean particle size of soybean meal was 1.5 mm and the levels of other parameters were fixed at the center of FFD, as shown in Table 3. To move away from the center of FFD as the origin point of the steepest ascent path, the basic step sizes of variables were considered 0.25, 0.33, 0.4, and 0.3 units of B, D, E, and G, respectively. The maximum production of rhamnolipids was seen at the run three (Table 3).

**Table 3:** Experimental design and results of the steepest ascent path

Run	Coded variables				Actual variables				Rhamnolipid (g/kg of substrate)
	B	D	E	G	B(%)	D(%)	E(°C)	G(mL)	
1	0	0	0	0	4	65	30	1	15.78
2	0.25	0.33	0.4	0.3	4.5	70	32	1.15	17.81
3	0.5	0.66	0.8	0.6	5	75	34	1.3	19.33
4	0.75	0.99	1.2	0.9	5.5	80	36	1.45	18.05
5	1	1.32	1.6	1.2	6	85	38	1.6	16.67
6	1.25	1.65	2	1.5	6.5	90	40	1.75	14.32

### Central composite design and response surface methodology

Following the optimization with the steepest ascent method, RSM using CCD was used to determine the actual optimum levels of significant factors and study the interactions. A total of 30 experiments were performed in duplicate; the levels of independent variables and experimental plans were given in Table 4.

**Table 4:** CCD design matrix with coded values along with the experimental and predicted rhamnolipid production

Run no	Independent variables				Rhamnolipid (g/kg of substrate)	
	A: Temperature (°C)	B: Inoculum size (mL)	C: Humidity (%)	D: Glycerol (%)	Experimental	Predicted
1	34.00 (0)	1.30 (0)	75.00 (0)	5.00 (0)	19.67	19.61
2	38.00 (+ α)	1.30 (0)	75.00 (0)	5.00 (0)	14.53	14.98
3	34.00 (0)	1.30 (0)	75.00 (0)	5.00 (0)	20.12	19.61
4	36.00 (1)	1.00 ( − 1)	80.00 (1)	6.00 (1)	16.1	15.89
5	36.00 (1)	1.60 (1)	80.00 (1)	4.00 ( − 1)	17.83	17.43
6	32.00 ( − 1)	1.60 (1)	80.00 (1)	4.00 ( − 1)	14.24	14.66
7	32.00 ( − 1)	1.00 ( − 1)	80.00 (1)	4.00 ( − 1)	16.42	16.13
8	34.00 (0)	1.30 (0)	75.00 (0)	5.00 (0)	19.11	19.61
9	32.00 ( − 1)	1.00 ( − 1)	70.00 ( − 1)	6.00 (1)	13.97	14.38
10	34.00 (0)	1.30 (0)	75.00 (0)	5.00 (0)	19.56	19.61
11	32.00 ( − 1)	1.00 ( − 1)	80.00 (1)	6.00 (1)	15.02	15.02
12	36.00 (1)	1.60 (1)	70.00 ( − 1)	6.00 (1)	16.25	16.55
13	32.00 ( − 1)	1.60 (1)	80.00 (1)	6.00 (1)	17.43	17.62
14	32.00 ( − 1)	1.60 (1)	70.00 ( − 1)	4.00 ( − 1)	14.42	14.64
15	32.00 ( − 1)	1.60 (1)	70.00 ( − 1)	6.00 (1)	18.13	17.78
16	34.00 (0)	1.30 (0)	75.00 (0)	7.00 (+ α)	15.12	15.13
17	34.00 (0)	1.30 (0)	65.00 ( − α)	5.00 (0)	16.78	17
18	30.00 ( − α)	1.30 (0)	75.00 (0)	5.00 (0)	13.68	13.4
19	34.00 (0)	1.30 (0)	75.00 (0)	5.00 (0)	19.53	19.67
20	34.00 (0)	1.30 (0)	75.00 (0)	5.00 (0)	19.71	19.67
21	36.00 (1)	1.00 ( − 1)	70.00 ( − 1)	6.00 (1)	13.81	13.19
22	36.00 (1)	1.60 (1)	70.00 ( − 1)	4.00 ( − 1)	15.55	15.04
23	34.00 (0)	1.30 (0)	75.00 (0)	3.00 ( − α)	14.88	15.4
24	36.00 (1)	1.00 ( − 1)	70.00 ( − 1)	4.00 ( − 1)	16.24	16.06
25	34.00 (0)	1.90 (+ α)	75.00 (0)	5.00 (0)	17.89	17.67
26	36.00 (1)	1.60 (1)	80.00 (1)	6.00 (1)	18.42	18.46
27	34.00 (0)	0.70 ( − α)	75.00 (0)	5.00 (0)	15.4	15.31
28	34.00 (0)	1.30 (0)	85.00 (+ α)	5.00 (0)	19.76	19.71
29	36.00 (1)	1.00 ( − 1)	80.00 (1)	4.00 ( − 1)	18.79	18.94
30	32.00 ( − 1)	1.00 ( − 1)	70.00 ( − 1)	4.00 ( − 1)	15.55	15.31

Empirical data were fitted by following second order polynomial equation:

3 $$\begin{aligned} {\text{Y}} = & - {19}.{62} + 0.{4}0{\text{A}} + 0.{\text{47B}} + 0.{\text{68C}} + 0.0{\text{24D}} - {9}.{\text{37E}}00{\text{3AB}} + 0.{\text{52AC}} - 0.{\text{48AD}} - 0.{\text{2BC}} + {1}.0{\text{2BD}} \\ & - 0.0{\text{44CD}} - {1}.{\text{36A}}^{{2}} - 0.{\text{72B}}^{{2}} - 0.{\text{31C}}^{{2}} - {1}.{\text{13D}}^{{2}} \\ \end{aligned}$$

where Y was predicted response, A, B, C and D were coded values of process temperature, inoculum size, initial humidity, and concentration of glycerol, respectively.

Analysis of Variance (ANOVA) was used to analyze the results and determine the significance of factors affecting the rhamnolipid production process. ANOVA for obtained response surface model was presented in Table 5. The model F-value of 52.80 and the P-value of < 0.0001 indicated model significance. The coefficient of determination (R^2^) was 0.9801 indicated that presented model appropriately fit the empirical data. The predicted R^2^ (0.9034) and adjusted R^2^ (0.9615) confirmed the model's suitability to predict the rhamnolipid amount produced as a function of model parameters. The lack of fit P-value of 0.2499 implied the lack of fit was not significant relative to the pure error. The greater significance of the component is shown by a lower P-value [31]. Among independent variables, A (temperature), B (inoculum size), and C (humidity) had significant effects on rhamnolipid production as their P-values were lower than 0.05. The quadratic term of four factors and the interactions between B and C, B and D also C and D were significant.

**Table 5:** ANOVA for the response surface quadratic model

Source of variation	Sum of square	*df*	Mean square	F	P-value	FI
Model	125.42	14	8.96	52.80	< 0.0001	Significant
A	3.77	1	3.77	22.21	0.0003	
B	5.37	1	5.37	31.63	< 0.0001	
C	11.06	1	11.06	65.16	< 0.0001	
D	0.014	1	0.014	0.080	0.7814	
AB	1.4E^−003^	1	1.4E^−003^	8.288E^−003^	0.9287	
AC	4.25	1	4.25	25.07	0.0002	
AD	3.75	1	3.75	22.12	0.0003	
BC	0.64	1	0.64	3.75	0.0719	
BD	16.59	1	16.59	97.75	< 0.0001	
CD	0.032	1	0.032	0.19	0.6727	
A^2^	50.44	1	50.44	297.30	< 0.0001	
B^2^	14.26	1	14.26	84.07	< 0.0001	
C^2^	2.72	1	2.72	16.03	0.0012	
D^2^	35.17	1	35.17	207.29	< 0.0001	
Residual	2.535	15	0.17			
Lack of fit	2.01	10	0.20	0.58	0.2499	Not Significant
Pure error	0.53	5	0.11			
Cor total	108.73	29				

R-square = 0.9801; Adj. R-square = 0.9615; Predicted R-square = 0.9034

### Effect of significant factors on rhamnolipid production

**Fig. 2:**
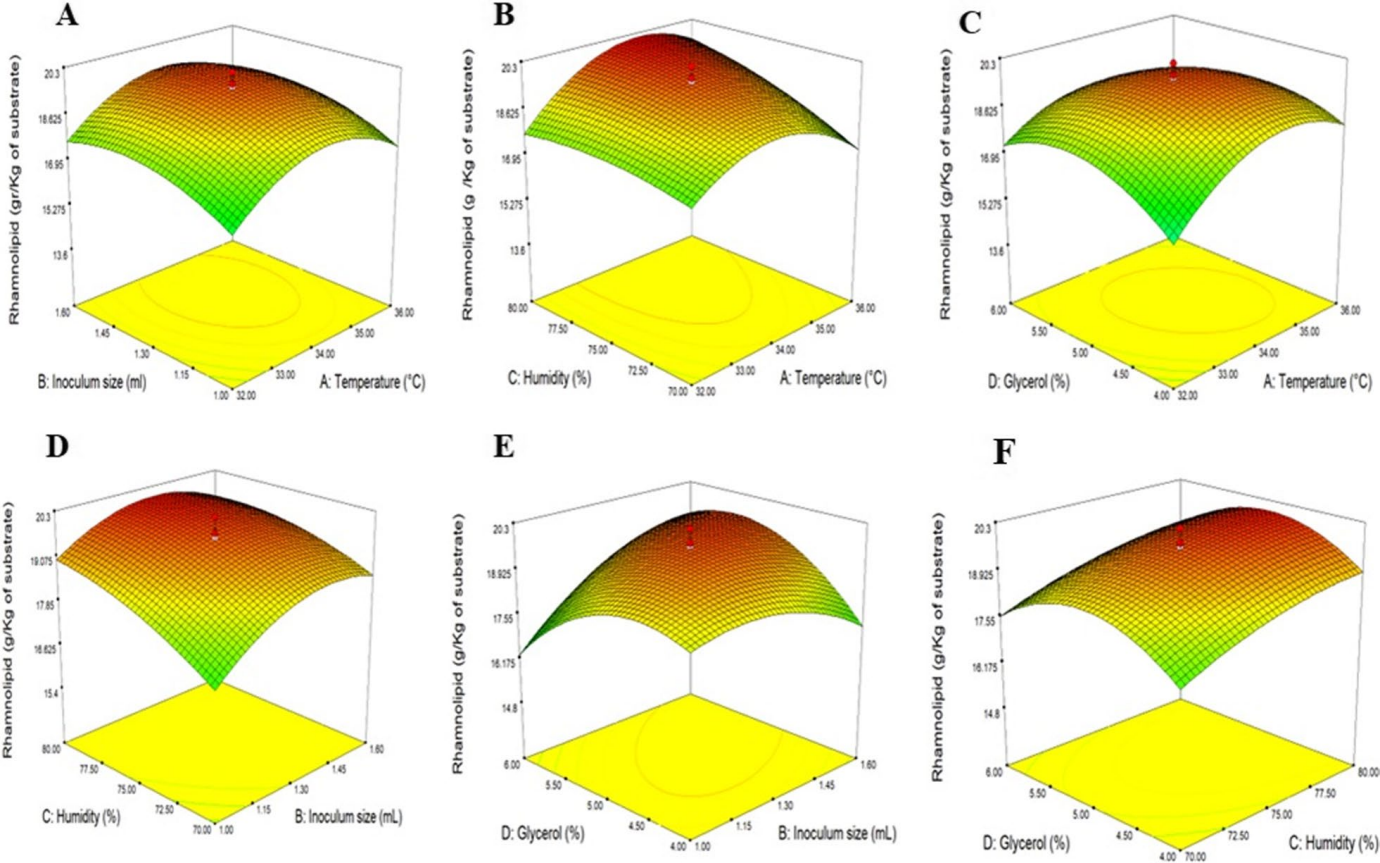
Response surface plots for rhamnolipid production: **A** interaction of temperature and inoculum size, **B** interaction of temperature and humidity, **C** interaction of inoculum size and humidity, **D** interaction of temperature and glycerol concentration, **E** interaction of inoculum size and humidity, **F** interaction of humidity and glycerol concentration

The three-dimensional (3D) graphs of response surfaces were plotted to explain factors' interactions and determine their optimum values to achieve the maximum biosurfactant production. Each figure illustrates two independent variables' effect while the other variables were held at their central values. Convex natures of 3D response surface curves represented in Fig. 2, indicate that the optimum conditions were well defined. Figure 2 showed that the interactions between temperature and inoculum size, humidity, and inoculum size, humidity, and the glycerol percentage were not significant. In contrast, the other two-factor interactions had significant effects.

In contrast to glycerol content, the ANOVA and 3D curve findings showed that the substrate's temperature, inoculum size, and starting humidity were crucial factors in the production of the biosurfactant. One of the most significant and deciding factors in bioprocesses is temperature, and each bacterial species has a different preferred temperature range. Due to Fig. 2A and B, the highest rhamnolipid production by *Pseudomonas aeruginosa* PTCC 1074 was obtained at the range of 34–35 °C. Since the temperature affects the biochemical reactions in microorganism cells, rhamnolipids production considerably changed at different temperatures [32].

Inoculum size plays an essential role to produce the biosurfactants in SSF. ANOVA results indicated that inoculum size had a considerable effect on the rhamnolipid production. The maximum production of rhamnolipid was obtained when 1.4 mL inoculum of *P. aeruginosa* was used. Regarding Fig. 2A, C, and E, rhamnolipids production was low for small inoculum sizes because a small number of bacterial cells in culture medium required more time to grow to the optimum number to use the substrate and formation of product. In general, increasing the inoculum size up to a certain point enhances the development of microorganisms and consequently growth-related activities, whereas growing it further decreases microbial activity since there are only so many nutrients available [33].

The humidity of substrate is other critical parameter in SSF to produce the value-added products. This factor is important because microbes grow and produce products on or near the surface of solid substrates containing moisture. At low substrate humidity levels, nutrient solubility reduces, whereas, at high humidity levels, substrate porosity or air content may decrease [34]. Under both conditions, biosurfactant production would decline compared to the optimal level of humidity. The optimal humidity of substrate for the biosurfactant production was 80%.

Since glycerol can act as a co-carbon source, rhamnolipid production improved when glycerol content increased up to 5% (v/v) in saline solution, it declined at higher glycerol contents. This result is in line with those of previous studies [14].

### Experimental validation of the optimized condition

The model predicted that the optimal values of significant variables were the temperature of 34.5 °C, inoculum size of 1.4 mL, the humidity of 80%, and glycerol content of 5% (v/v), which were obtained by solving the regression equation using the numerical optimization function in the Design-Expert software. Under optimal circumstances, three further tests were conducted to assess the accuracy of the model in predicting the maximal rhamnolipids production. The rhamnolipid output's mean value was 19.68 g/kg dry substrate, which was in good agreement with the model predicted value (20.13 g/kg dry substrate). Since the kinetics of biosurfactant production was studied in non-optimal conditions, rhamnolipid production under optimal conditions was measured once again for different incubation periods (Fig. 1A). The results showed that the biosurfactant production was increased by 34% in the optimized, when compared to that in unoptimized conditions and proved that the model was appropriate.

### Structural characterization

The detection of TLC plate with iodine confirmed the presence of di and mono- rhamnolipid with the *R*_*f*_ values of 0.35 and 0.76. A similar *R*_*f*_ value of 0.38 for di-rhamnolipid and 0.85 for mono-rhamnolipids was observed by Nalini and Parthasarathi [27].

**Fig. 3:**
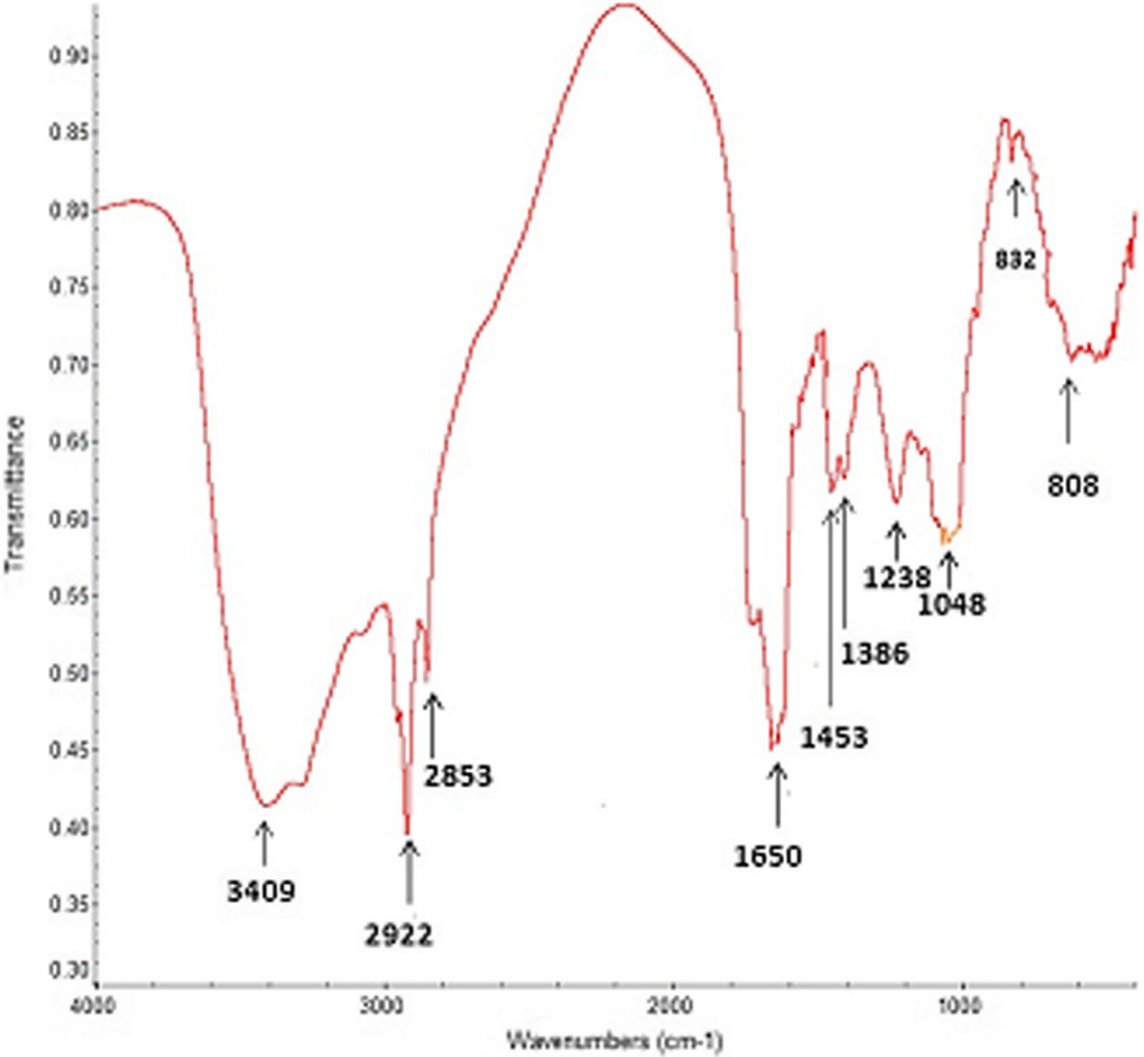
FT-IR spectrum of rhamnolipid showing the following vibrations: O–H stretching (3409 cm^−1^), C–H stretching asym. (2922 and 28,563 cm^−1^), C=O stretching (1650 cm^−1^), C–H deformations (1453, 1238 and 808 cm^−1^), C–H/O–H deformation (1386 cm^−1^), C–O stretching (1048 cm^−1^), α-pyranyl II sorption band (832 cm.^−1^)

Figure 3 illustrates FT–IR spectrum of purified biosurfactant from *P. aeruginosa*. The broad peak at 3409 cm^−1^ revealed the presence of O–H stretching vibrations. The absorption band at 2922 cm^−1^ showed the asymmetric C–H stretch of CH_2_ and CH_3_ groups of aliphatic chains. The corresponding symmetric stretch was seen at 2853 cm^−1^. The peaks next to 1650 cm^−1^ were assigned to C=O stretching in protein structure. The fingerprint areas between 400 cm^–1^ and 1500 cm^–1^ indicated the C–H deformations at 1453 and 1238, the C–OH deformation at 1386 cm^–1^, and the symmetric band at 1048 cm^–1^. The spectrum showed α-pyranyl II sorption band at 832 cm^−1^, which confirmed the presence of di-rhamnolipid in the mixture. The results of IR spectra indicated the presence of rhamnose rings and hydrocarbon chains in the chemical structure of obtained biosurfactants and are identical with the report of Guo et al. [35].

**Fig. 4:**
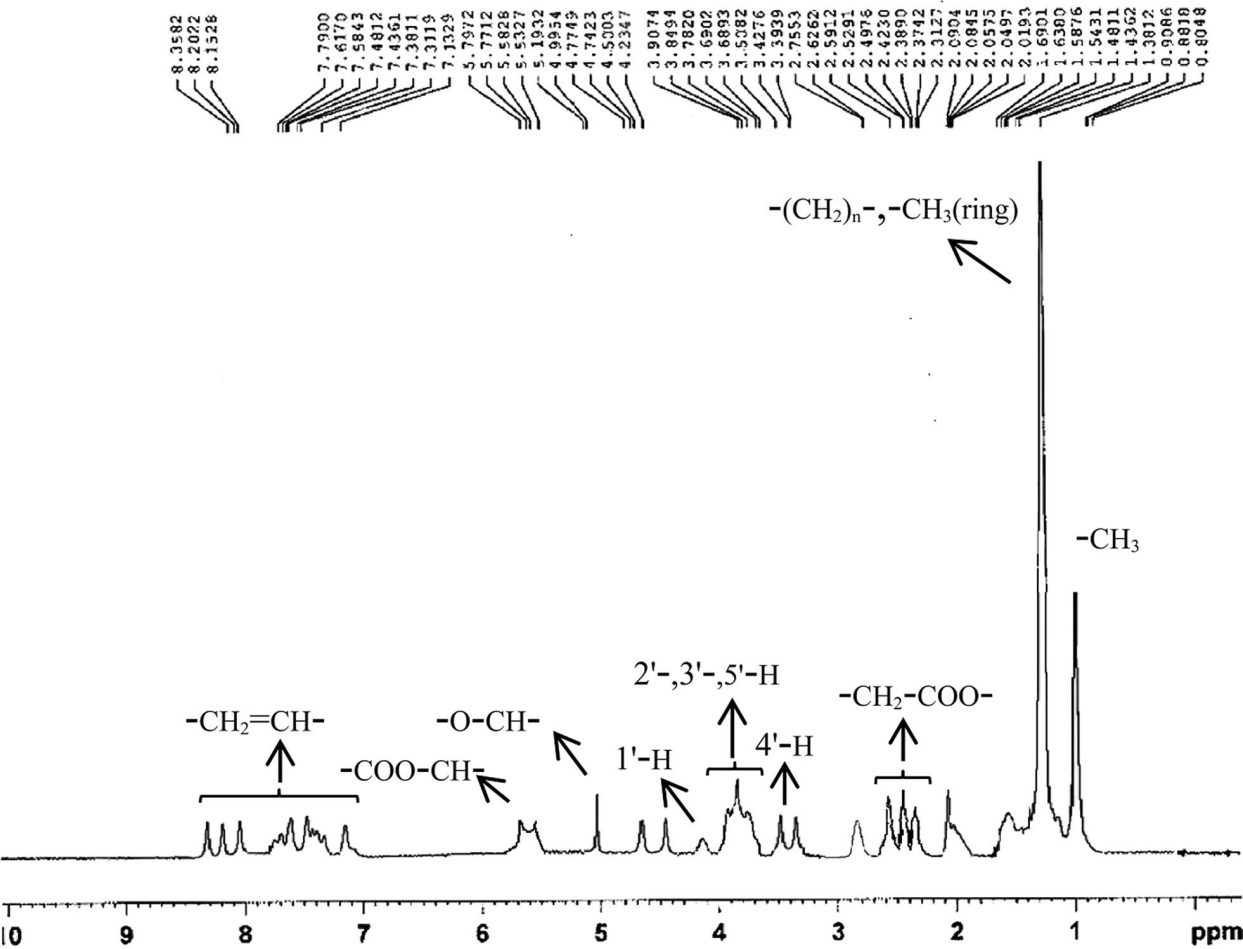
^1^H-NMR spectrum of produced biosurfactant by *Pseudomonas aeruginosa* PTCC 1047

According to the results of ^1^H-NMR spectrum (Fig. 4), the characteristic chemical shifts confirmed that the fermentation product was a mixture of two forms of rhamnolipids and had functional groups, bonds and structures which are presence in rhamnolipid type biosurfactants. The chemical shifts at 0.88 ppm indicated the presence of CH_3_ and characteristic chemical shifts at 1.38, 2.48, 4.99, and 5.57 ppm showed the presence of –(CH_2_)n–, –CH_2_COO–, –OCH–, and –COO-CH– groups respectively. The results were comparable to the previous reports [36, 37].

### Properties of the produced biosurfactant

#### Determination of critical micelle concentration (CMC)

Commonly used to define the surfactant efficiency is CMC, a crucial parameter of any surface-active chemical. Because less surfactant is needed for saturating surfaces and the production of micelles, efficient surfactants have low CMC values. The surface tension values were measured in a wide range of rhamnolipid concentrations (0–100 mg/L), and results (Fig. 1B) showed that by increasing rhamnolipid concentration up to 70 mg/L, surface tension was reduced from 71.8 ± 0.4 to 32.2 ± 0.2 mN/m and then remained relatively constant. Hence, the value of 70 mg/L was determined for the CMC of produced rhamnolipid, which is very lower than the CMC of sodium dodecyl sulphate, a prevalent synthetic surfactant (2347 mg/L) [38, 39].

CMC value obtained in this study is in conformity with the results of previous reports using agro-industrial residues to produce biosurfactants [40].

### Stability analysis

**Fig. 5:**
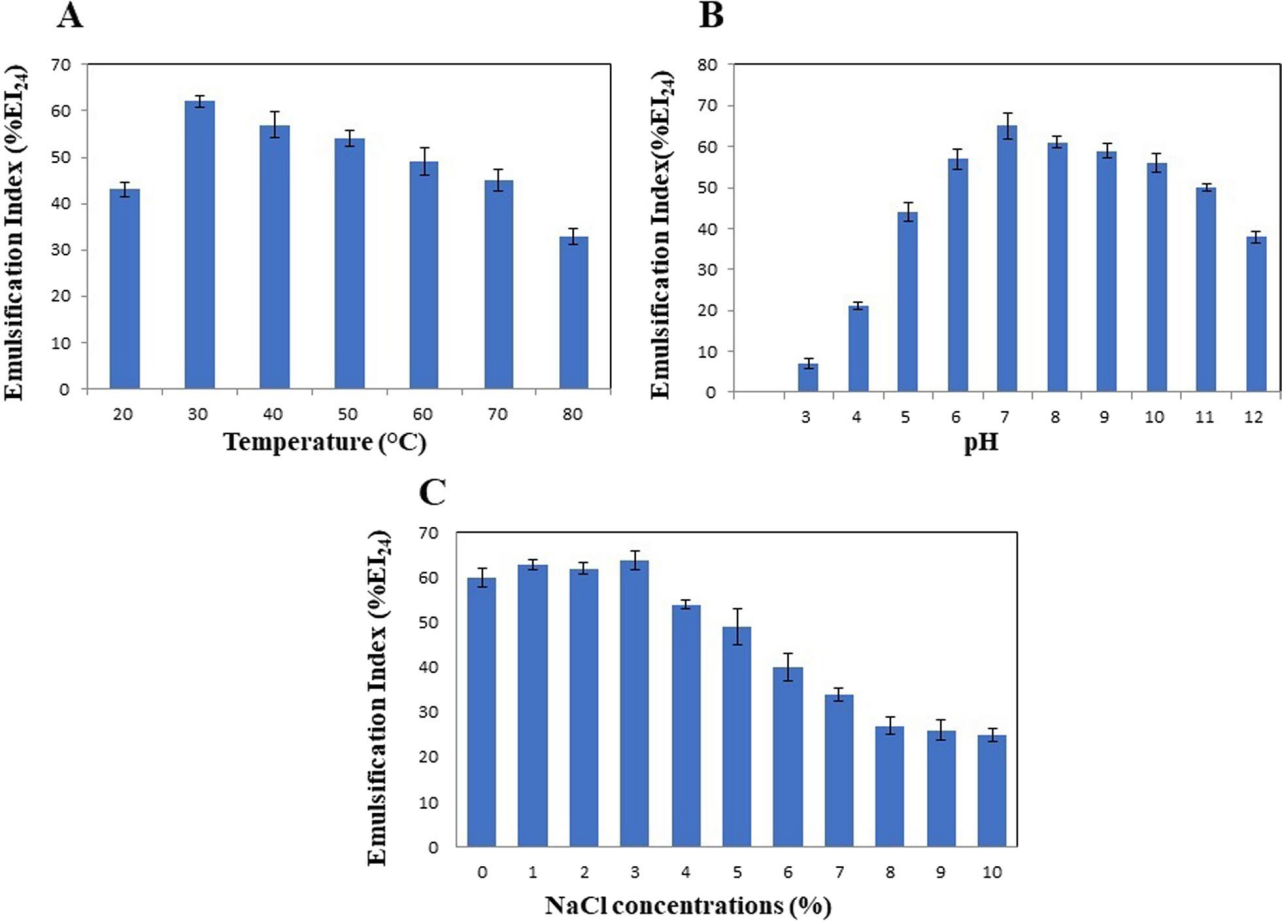
Stability studies of rhamnolipid under different temperature (**A**), pH (**B**), and salinity (**C**)

Biosurfactants' stability in different environmental conditions is one of the most important factors for their application in various fields. The biosurfactants' stability at different temperatures, pH values, and salt concentrations was evaluated using %EI_24_. Due to the results (Fig. 5A), the highest extent of emulsification was observed at 30 °C. Increasing temperature to 70 °C had no significant effect on rhamnolipid yields. Thermal stability in the range of 30–70 °C is a valuable attribute of generated rhamnolipid in these industries, since thermal processing is employed in these sectors to establish sterile conditions [41]. Regarding pH effect, the highest %EI_24_ was obtained at pH 7, and no significant decrease was observed in %EI_24_ at pH 6–10, but the index significantly decreased at pH 3–4 (Fig. 5B). In other words, the rhamnolipid biosurfactant was more stable at basic pH than at acidic pH. It can be attributed to the precipitation of anionic biosurfactants such as rhamnolipid at low pH values and the greater stability of fatty acid surfactant micelles at high pH values. Similar results were reported in previous studies [41, 42].

Evaluating the biosurfactant stability at different sodium chloride concentrations, presented in Fig. 5C, showed that the highest stability pertained to the concentration of 1% (w/v), and increasing the concentration to 6% (w/v) did not cause a significant change in %EI_24._ It indicated that the biosurfactant was stable in a suitable concentration range. Due to these results, the produced rhamnolipid could be used in different fields [43].

## Conclusions

Results showed the potential use of soybean meal as substrate for the production of rhamnolipids biosurfactant using Pseudomonas aeruginosa PTCC 1074 under SSF. Screening the key nutritional and environmental parameters indicated that glycerol concentration, humidity, temperature, and inoculum size strongly affected rhamnolipid production. The biosurfactant production was increased by 34% in the optimized, when compared to that in unoptimized conditions. The quadratic model's suitability and accuracy were established by validation trials, and the findings demonstrated that the projected values and experimental data agreed well. Rhamnolipids were found in the biosurfactant after being characterized by TLC, FT-IR, and 1H-NMR.Rhamnolipid biosurfactant represented high surface activity and good stability over a wide range of temperatures, pH, and sodium chloride concentrations, making it a potential candidate for use in different applications.

## Methods

### Microorganism

*Pseudomonas aeruginosa* (PTCC 1074), a potent biosurfactant producer, was obtained from Persian Type Culture Collection (PTCC). The strain was maintained on nutrient agar slants at 4 °C and subculture before using inoculums for biosurfactant production. Then, a loop of cells was transferred into 50 mL LB broth in 250 mL Erlenmeyer flask and incubated at 30 °C until the growth of *Pseudomonas aeruginosa* achieved mid-exponential phase at an optical density of 0.6 to 0.8 at 600 nm. Then, this culture was utilized as inoculum for SSF.

### Substrate

Soybean meal was obtained from the local market and grinned in a mixer grinder and passed through the standard sieves No.35 and No.10 with a mean particle size of 0.5 and 1.5 mm, respectively, washed, dried, and stored until further use.

### Production of biosurfactant by SSF

Fermentation experiments were carried out in a 250 mL Erlenmeyer flask containing 5 g of soybean meal. To do it, a salt solution and various amounts of water were added to obtain the desired humidity. The salt solution consisted of (g/l): KH_2_PO_4_ 3, K_2_HPO_4_ 7, different amount of MgSO_4_.7H_2_O, NaNO_3_, and glycerol (v/v %). Then, flask was sterilized in an autoclave for 15 min at 121 °C, and after cooling to room temperature, different amounts of inoculum of *Pseudomonas aeruginosa* were added. The inoculated flasks were incubated at various temperatures due to the designed experiment runs for 240 h.

### Biosurfactant extraction

Acid precipitation and liquid–liquid extraction methods were used to extract biosurfactants from SSF. Each SSF flask received 50 mL of distilled water and was agitated for 1 h at 200 rpm at 30 °C. Then, obtained suspension has passed through cheesecloth, and excess liquid was squeezed out. This procedure was carried out triplicate, and the extract was then centrifuged for 15 min at 10,000 × g. The supernatants' pH was adjusted to approximately 2 with 2 N HCl, and biosurfactants were extracted three times with chloroform–methanol (2:1, v/v). The organic phase was concentrated in a vacuum evaporator, and the obtained biosurfactant was stored for further analysis.

### Quantification of rhamnolipids

The concentration of extracellular rhamnolipids was evaluated in triplicate by quantifying rhamnose concentration using the orcinol method. The extracted biosurfactant was dissolved in water, and 50 μL of this sample, was mixed with 450 μL of orcinol reagent (0.19% orcinol in 53% sulphuric acid). The formed mixture was heated at 80 °C for 30 min and cooled to room temperature. The rhamnose content was determined by measuring the mixture's absorbance at 421 nm and comparing the data with a standard curve prepared using different L-rhamnose [44]. The rhamnose moiety constitutes only part of rhamnolipid molecule; therefore, rhamnolipid concentration is obtained by multiplying rhamnose content by a correction factor ranging from 3.0 to 3.4 [45, 46]. An average value of 3.2 was considered.

### Design of experiment

#### Two-level fractional factorial design

At the first optimization step, a two-level fractional factorial design was employed to identify which process parameter significantly affects rhamnolipid production. Eight major factors pH, concentration of glycerol (%), amount of MgSO_4_.7H_2_O (g), humidity (%), temperature (°C), size of substrate (nm), inoculum size (mL) and amount of NaNO_3_ (g)); were studied at two levels, high (+ 1) and low ( − 1), using 2^8–4^ fractional factorial design. Table 2 lists the factors and their levels in experimental design. A total of 16 experimental runs were performed in duplicates to complete the design, and biosurfactant's production was measured as a response variable. Table 2 also shows the fractional factorial design and corresponding observed and predicted results. Considering regression analysis, factors with a P-value lower than 0.05 statistically have a significant effect on biosurfactant production. P-values were used as a suitable method to examine the importance of model parameters, which was required to comprehend the pattern of reciprocal interactions among the most important elements.

#### Path of the steepest ascent

As FFD can't predict the actual optimum values of variables, the method of steepest ascent was employed to move rapidly to the region of optimum operating conditions. In the steepest ascent experiments, the main variables moved in the directions of maximum increase in the response. In this way, the steps through the steepest ascent path were proportionate to regression coefficients gained from FFD and the experiments were performed until no further increases in the response were observed. This point is close to the optimal point which could be considered a center point for optimization [47].

#### Central composite designs (CCD) or RSM

Due to the screening results with FFD, the factors with significant effects on biosurfactant production and their interaction effects were analyzed and optimized by RSM using a CCD. CCD was widely used as an effective method for fitting multivariate nonlinear equations to optimize process variables [30]. For four factors, CCD was made up 2^4^ runs at factorial points, consisting of possible combinations of + 1 and − 1 levels of the factor, augmented with six replicate runs at the center point and eight runs at axial points, which have one factor at an axial distance (α) from the center. In contrast, the other factor is at level 0. To obtain a rotatable design, a value of 2 was considered for axial distance (α). The response surface regression procedure was applied to analyze the experimental results. A second-order polynomial equation for correlation among independent variables which the response can be presented as follows:

4 $$\begin{aligned} {\text{Y}} = & \, \beta_{0} + \, \beta_{{1}} {\text{A }} + \, \beta_{{2}} {\text{B }} + \, \beta_{{3}} {\text{C }} + \, \beta_{{4}} {\text{D }} + \, \beta_{{{11}}} {\text{A}}^{{2}} + \, \beta_{{{22}}} {\text{B}}^{{2}} + \, \beta_{{{33}}} {\text{C}}^{{2}} + \, \beta_{{{44}}} {\text{D}}^{{2}} \\ & + \, \beta_{{{12}}} {\text{AB }} + \, \beta_{{{13}}} {\text{AC }} + \, \beta_{{{14}}} {\text{AD }} + \, \beta_{{{23}}} {\text{BC }} + \beta_{{{24}}} {\text{BD }} + \, \beta_{{{34}}} {\text{CD}} \\ \end{aligned}$$

where Y is predicted response, β_0_ is offset term; β_1_, β_2,_ β_3_, and β_4_ are linear coefficients; β_11_, β_22_, β_33_, and β_44_ are quadratic coefficients; β_12_, β_13_, β_14_, β_23_, β_24_ and β_34_ are interaction coefficients; A, B, C and D are independent variables. The “Design Expert 7.0” software was applied to experimental data analysis and to obtain response surface curves to optimize the variables.

#### Characterization of the biosurfactant

##### Thin layer chromatography (TLC)

The obtained biosurfactant was analyzed by TLC using silica gel 60 G (Merck) and a solvent mixture of chloroform–methanol–water (65:15:2, v/v/v). The spots were detected by iodine reagent, and *R*_*f*_ value of each macromolecule was noted using the following formula [48]:

5 $$R_{f} = {\text{ Distance}}\;{\text{ travelled}}\;{\text{ by }}\;{\text{substance}}/{\text{Distance}}\,{\text{ travelled}}\;{\text{ by}}\;{\text{ the}}\;{\text{ solvent}}.$$

##### Fourier transform infrared spectroscopy (FTIR)

The FT-IR spectrum of crude biosurfactant was recorded using KBr pellet as a background reference in JASCO 4600 FTIR spectrophotometer. IR spectra were reported in 500–4000 wave numbers (cm^−1^).

##### Nuclear magnetic resonance spectroscopy (NMR)

For the NMR analysis, the produced biosurfactant was re-dissolved in deuterated chloroform, and ^1^H spectra was measured using a Bruker Avance DRX 500 MHz spectrometer.

### Properties of produced biosurfactant

#### Determination of critical micelle concentration (CMC)

Du-Nouy ring method was used to measure CMC by Tensiometer (Kruss K6, Germany) [49]. In this method, a platinum ring is submerged in the liquid and then slowly removed. The force required to remove the ring from liquid surface is considered as the surface tension. Surface tension was measured at different biosurfactant concentrations, and the plot of surface tension vs. concentration was obtained. CMC represents the concentration at which micelles start, and a sudden drop in surface tension is observed [41].

#### Stability study

Stability studies were evaluated regarding environmental conditions using the Emulsification Index (% EI_24_). To measure % EI_24_, 2 mL of cell-free supernatant was added to 2 mL of kerosene, and the mixture vortexed for 2 min. After 24 h, % EI_24_ was calculated using following equation [50]: Eq. (6)

6 $${\text{\% EI}}_{24} = \frac{{\text{Height of emulsified layer}}}{{{\text{Height of total liquid }}\left( {{\text{sum of aqueous}},{\text{ kerosene and emulsified layer}}} \right)}} \times 100$$

To investigate the thermal stability of produced biosurfactant, cell-free supernatant was stored at constant temperatures of 20, 30, 40, 50, 60, 70, and 80 °C for 30 min. Then, it was cooled to the room temperature (25 °C), and % EI_24_ was calculated. To determine biosurfactant stability at different pH values, the cell-free supernatant's pH was adjusted in the range of 3–12 using 1 N NaOH and 1 N HCl; % EI_24_ was measured after 30 min. To study the effect of sodium chloride on rhamnolipid biosurfactant, the purified biosurfactant was dissolved in distilled water containing various concentrations of NaCl (% w/v); % EI_24_ was determined after 30 min [51].

## Data Availability

All data generated or analyzed during this study are included in this published article.

## Abbreviations

EOR: Enhanced oil recovery
SSF: Solid state fermentation
FFD: Fractional factorial design
RSM: Response surface methodology
SMF: Submerged fermentation
PTCC: Persian type culture collection
CCD: Central composite designs
TLC: Thin layer chromatography
FTIR: Fourier transform infrared spectroscopy
NMR: Nuclear magnetic resonance spectroscopy
CMC: Critical micelle concentration
% EI_24_: Emulsification index
ANOVA: Analysis of variance
